# Supplementary material for: Determining the bacterial cell biology of Planctomycetes
Source: Nat Commun. 2017 Apr 10;8:14853. doi: 10.1038/ncomms14853 (PMC5394234; doi:10.1038/ncomms14853)
Supplement: Supplementary Information — Supplementary Figures, Supplementary Tables and Supplementary References [file ncomms14853-s1.pdf]

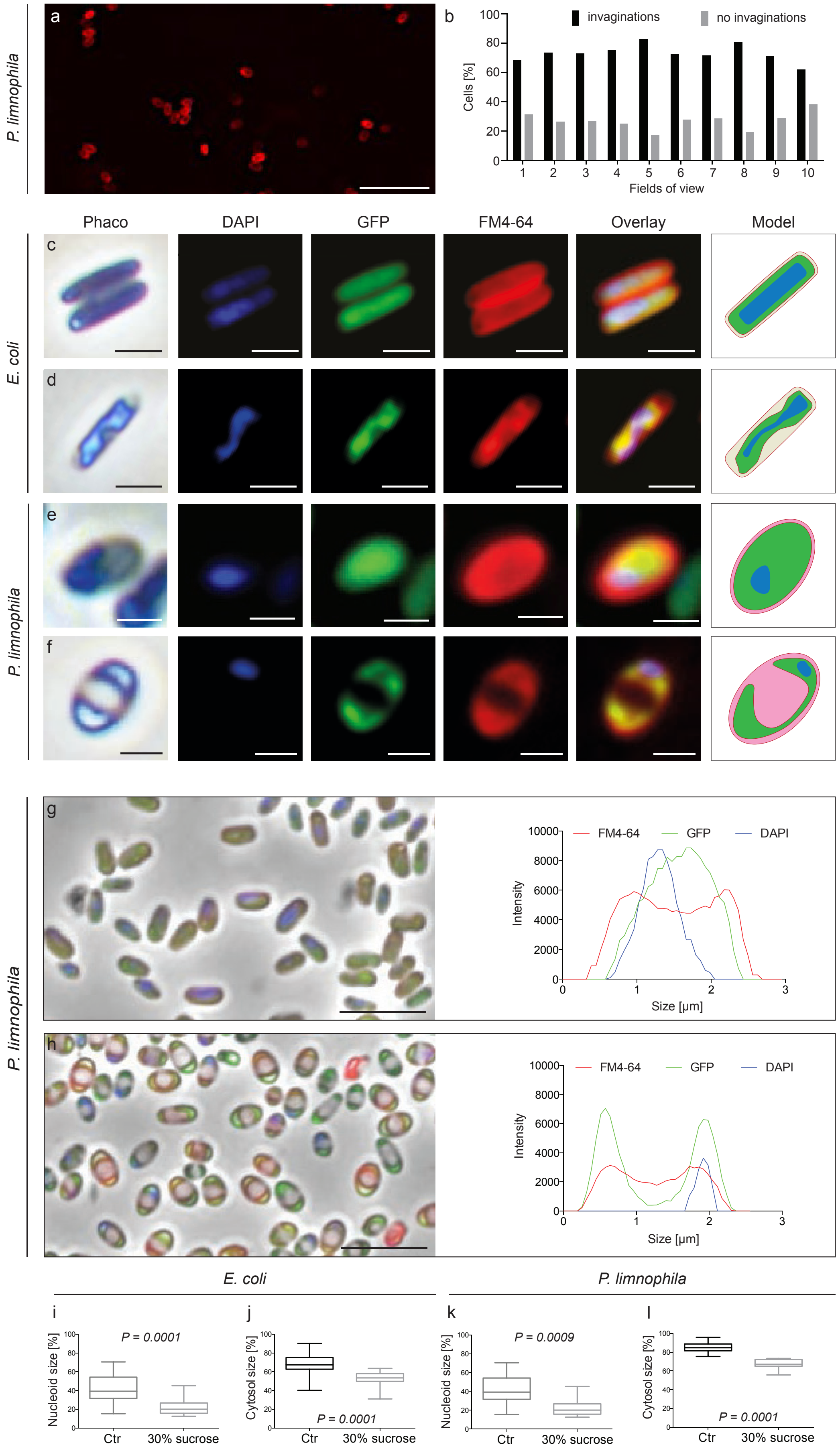

P. limnophila
a
b
invaginations
no invaginations
Cells [%]
Fields of view
Phaco
DAPI
GFP
FM4-64
Overlay
Model
E. coli
c
d
P. limnophila
e
f
P. limnophila
g
h
FM4-64
GFP
DAPI
Intensity
Size [µm]
E. coli
P. limnophila
i
j
k
l
Nucleoid size [%]
Cytosol size [%]
P = 0.0001
P = 0.0001
P = 0.0009
P = 0.0001
Ctr
30% sucrose

Supplementary Figure 1: Plasmolytic stress increases the cytoplasmic invaginations of *P. limnophila*

(**a+b**) Representative section of one field of view with Fm4-64 (red) stained cells. In (**b**) the ration between morphotype 1 (no invaginations) and morphotype 2 (invaginations) is given for 1838 cells among 10 randomly chosen fields of view. Scale bar: 10 µm.

(**c-f**) Artificial invagination of the cytoplasmic membrane by plasmolysis. GFP (green) expressing *E. coli* (**c+d**) and *P. limnophila* (**e+f**) cells were untreated (**c+e**) or osmotically stressed with 30% sucrose (**d+f**). *E. coli* cells form characteristic periplasmic bays (**d**, Phaco). Both, nucleoid (blue) and cytoplasm (green) of *E. coli* cells are shrunk while the membrane signal (red) resembles the staining pattern of morphotype 2 *P. limnophila* cells (**e** and Figure 2**c+d**). Such natural occurring cytoplasmic membrane invaginations of *P. limnophila* morphotype 2 cells (**e** and Figure 2**c+d**) increased by osmotic stress and became visible in phase contrast (**f**, Phaco). The cytoplasm shrunk further (**f**, GFP) and a black dot became visible in membrane staining experiments (**f**, FM4-64). Scale bars: 1 µm.

(**g+h**) Overlay of WF, Phaco, Fm4-64 (red), GFP (green) and DAPI (blue) signals of untreated (**g**) and treated (**h**) *P. limnophila* cells. The overview demonstrates that in (**e**) and (**f**) selected cells were representative. Corresponding fluorescence intensity profiles of untreated- (**g**) and osmotically stressed cells (**h**) revealed one cytosolic GFP maxima of the untreated control while a separation of the cytosolic GFP signal into two distinct intensity maxima was observed among treated cells (**h**). Scale bars: 5 µm.

(**i-l**) Quantification revealed a significant decrease of both, size of the nucleoid and size of the cytosol in *E. coli* (**i+j**) and *P. limnophila* (**k+l**), if treated with 30% sucrose compared to the respectively untreated control. (T-test: P=0.0001, P=0.0001, P=0.0009 and P=0.0001 respectively, error bars: min to max of 10 measured cells among 2 individual experiments).

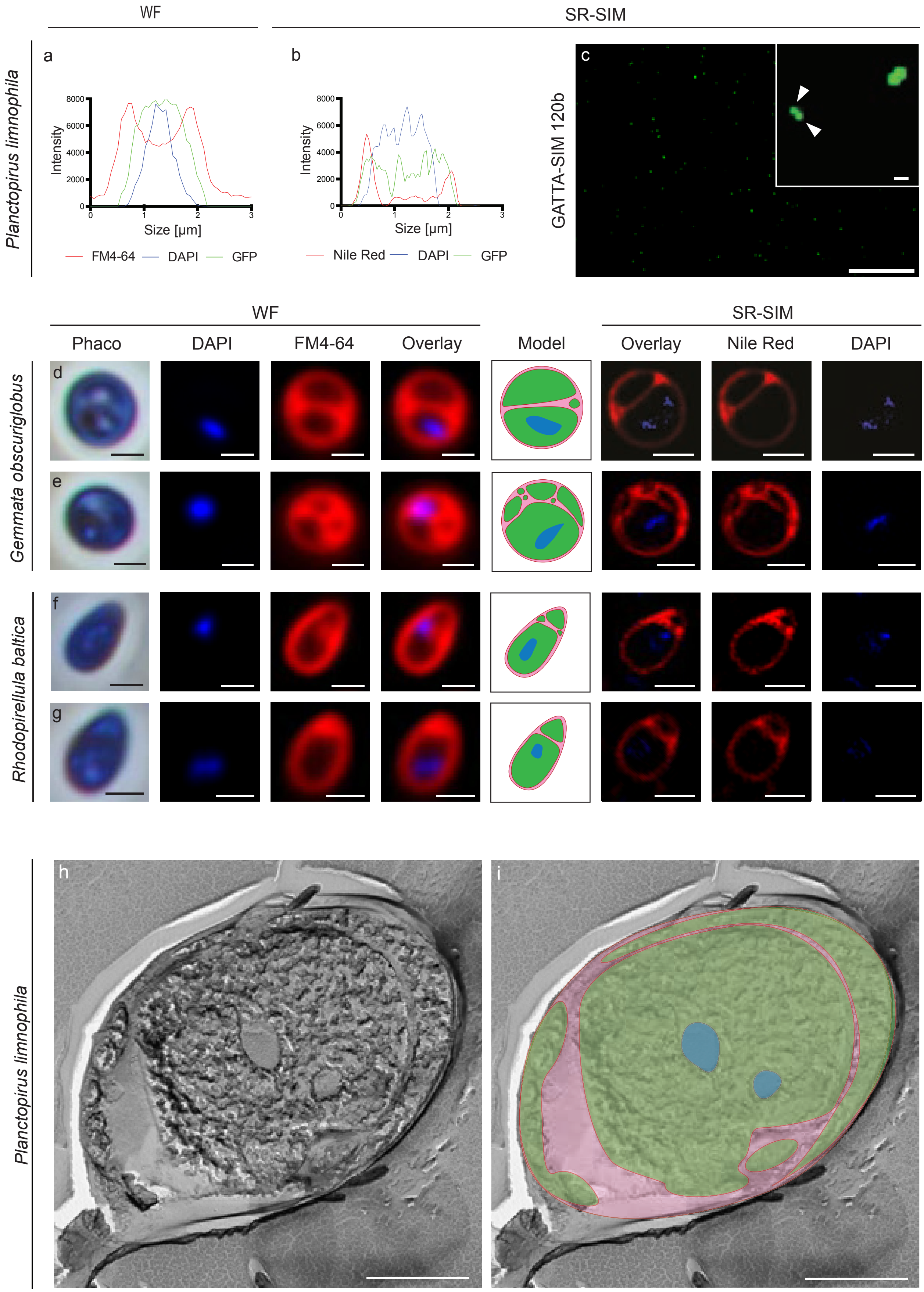

WF
SR-SIM
Planctopirus limnophila
a
b
c
Intensity
Size [µm]
FM4-64
DAPI
GFP
Nile Red
GATTA-SIM 120b
Phaco
DAPI
FM4-64
Overlay
Model
Nile Red
Gemmata obscuriglobus
Rhodopirellula baltica
d
e
f
g
h
i

Supplementary Figure 2: The innermost planctomycetal membrane is the cytoplasmic membrane

(**a-c**) Control of super resolution structured illumination (SR-SIM) performance under experimental conditions. (**a+b**) Representative fluorescence intensity line profiles of FM4-64 / Nile Red (red) and DAPI (blue) stained, GFP (green) expressing *P. limnophila* cells imaged in wide field- (**a**: WF) and SR-SIM (**b**). The width of outer- and inner membrane peaks (red) was 442 +/- 68 nm for WF- and 225 +/- 44 nm for SR-SIM experiments (20 randomly chosen cells). Thus, SR-SIM roughly doubled the resolution compared to WF. (**c**) Overview of a representative SR-SIM image showing multiple DNA origamis, each with two Alexa Fluor 488 dyes in 120 nm distance. Signals of both fluorophores overlap (**c**: magnification, white arrowheads), indicating that the resolution is >120 nm. Scale bars: 5 µm and 0.1 µm respectively.

(**d-g**) *G. obscuriglobus* (**d+e**) and *R. baltica* (**f+g**) cells analysed in WF and structured SR-SIM. Phase contrast (Phaco) microscopy revealed intracellular structures. In both species, the blue nucleoid (DAPI) is highly condensed and the red cytoplasmic membrane (FM4-64 / Nile Red) exhibit multiple invaginations as illustrated in the model. SR-SIM analyses resolved membrane invaginations that appear as red dots or lines in WF micrographs. Scale bars: 1 µm.

(**h+i**) A representative freeze fractured, plunge-frozen *P. limnophila* cell (**h**) with enlarged periplasm, caused by multiple invaginations of the cytoplasmic membrane (selected from 50 cells among 3 independent experiments). (**i**) False colours correspond to colours used in the models above (**d-g**). Scale bars: 0.5 µm.

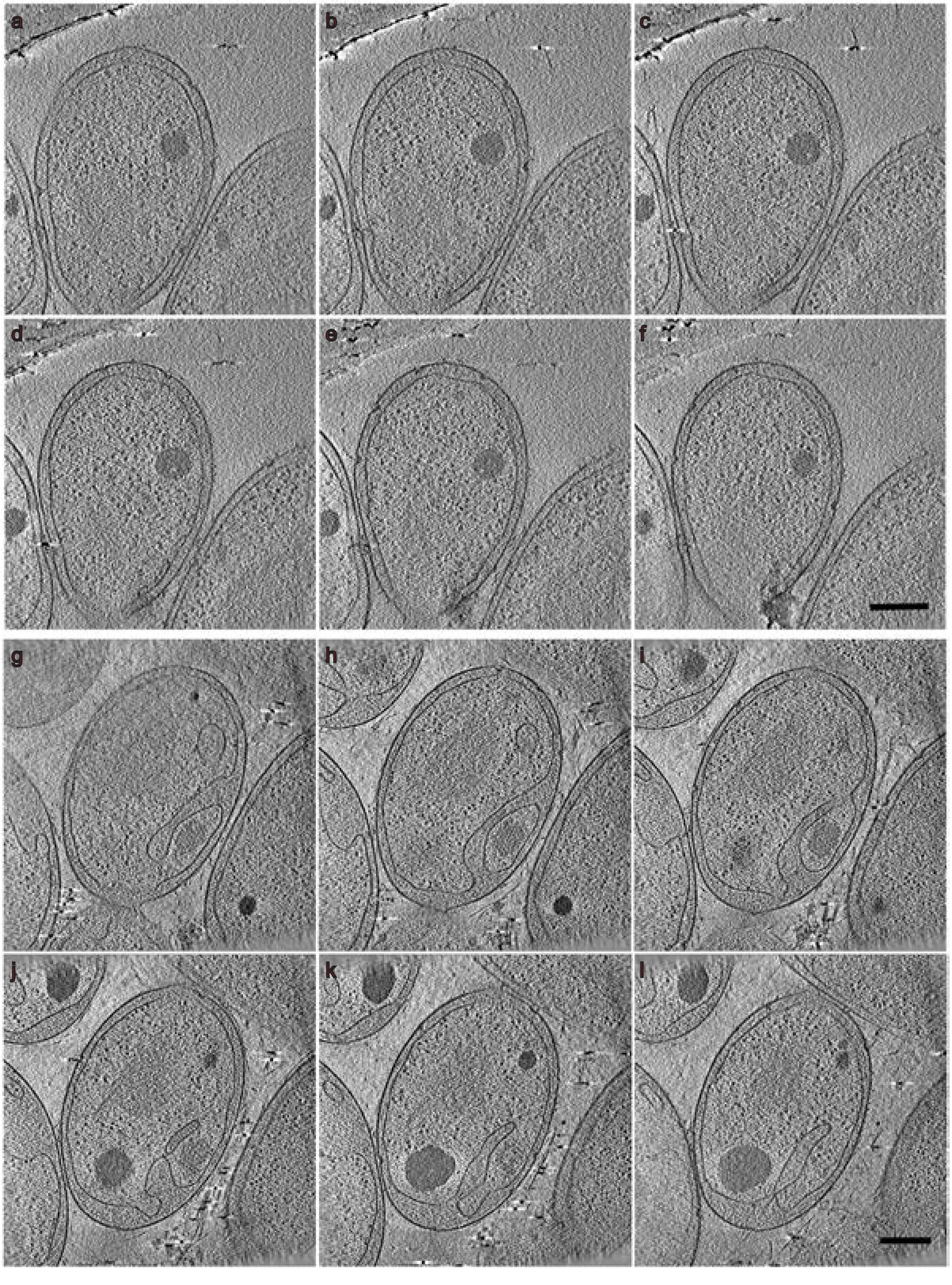
a
b
c
d
e
f
g
h
i
j
k
l

Supplementary Figure 3: Cryo-electron tomography of *Planctopirus limnophila* cells

(**a-f**) Example of a cell without invaginations of the cytoplasmic membrane. Representative slices of a tomogram of a FIB-thinned cell (thickness of remaining cell volume 260 nm; inter-slice distances are 17, 21, 17, 21 and 17 nm). (**g-l**) Cell with "tubular"- or "disk"-like invaginations apparently "enclosing" part of the cytoplasm (second image from top left). Slices are from a tomogram of a FIB-thinned cell (thickness of remaining cell volume 360 nm; inter-slice distances are 41, 38, 41, 38 and 38 nm). Scale bars: 250 nm.

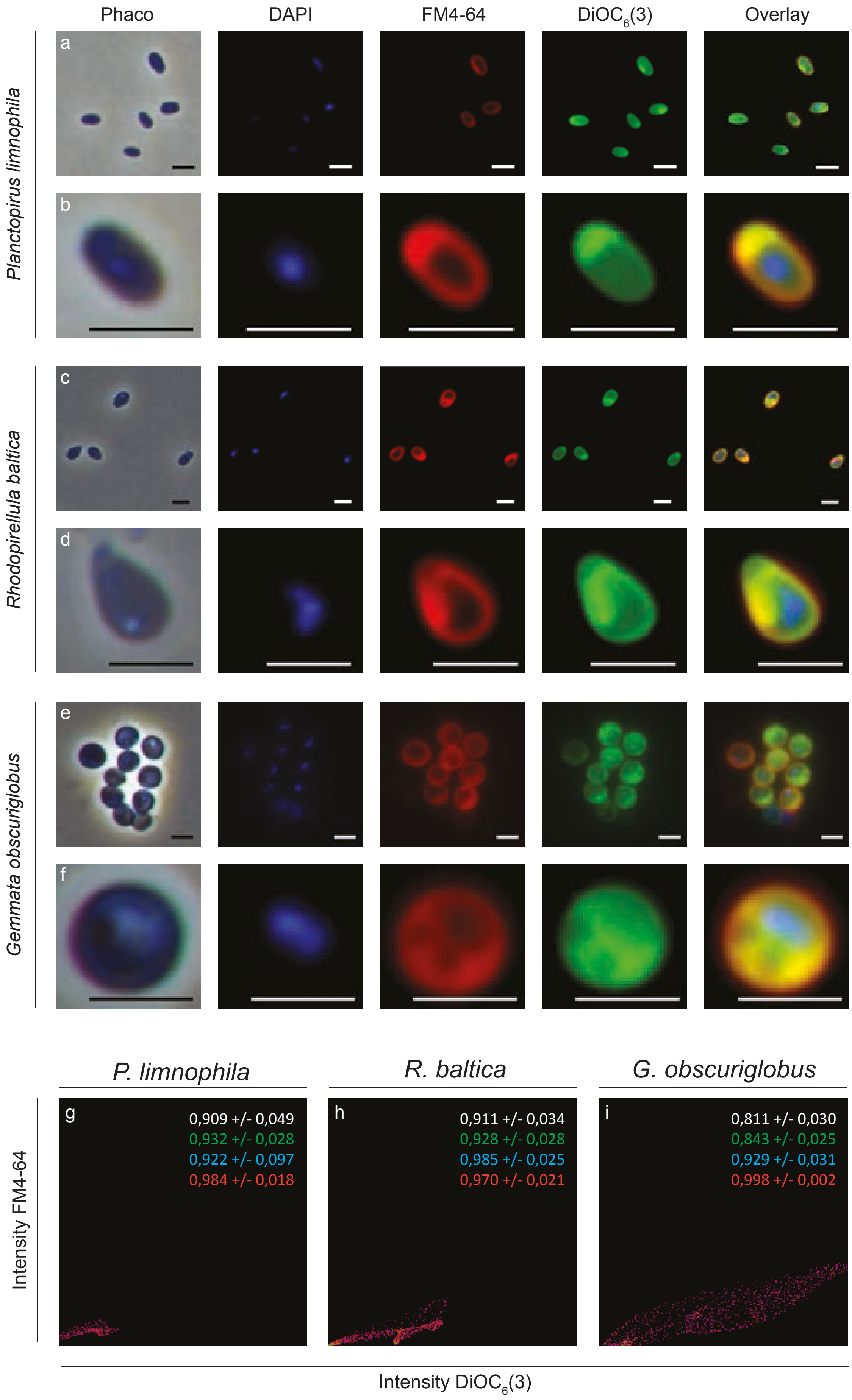
Phaco
DAPI
FM4-64
DiOC6(3)
Overlay
Planctopirus limnophila
Rhodopirellula baltica
Gemmata obscuriglobus
a
b
c
d
e
f
P. limnophila
R. baltica
G. obscuriglobus
g
0,909 +/- 0,049
0,932 +/- 0,028
0,922 +/- 0,097
0,984 +/- 0,018
h
0,911 +/- 0,034
0,928 +/- 0,028
0,985 +/- 0,025
0,970 +/- 0,021
i
0,811 +/- 0,030
0,843 +/- 0,025
0,929 +/- 0,031
0,998 +/- 0,002
Intensity FM4-64
Intensity DiOC6(3)

Supplementary Figure 4: Membrane potential at the innermost membrane

(**a-f**) The planctomycetal species, *P. limnophila* (**a+b**), *R. baltica* (**c+d**) and *G. obscuriglobus* (**e+f**) were stained with DAPI (DNA, blue), FM4-64 (membrane, red) and $DiOC_6(3)$ (energized membrane, green). The overlays show a distinct red signal around the outer rim of all three species. This indicates that the outermost membrane is not energized. The innermost membranes appear yellow in the overlays, which provides evidence that they are energized and correspond to the common bacterial cytoplasmic membrane. Scale bars: 2 µm.

(**g-i**) Intensity blots of FM4-64 versus $DiOC_6(3)$ signals reveal a typical linear correlation which is slightly blurry for *G. obscuriglobus* cells (**i**). Calculation of Pearsons's correlation (white), Mander's overlap (green) and co-localisation coefficients c1 (blue) and c2 (red) further support co-localisation of FM4-64 and $DiOC_6(3)$ signals among all analysed species. For (**g+h**) 50 individual cells were compared. For (**i**) 10 fields of view were used, due to aggregate formation of *G. obscuriglobus*.

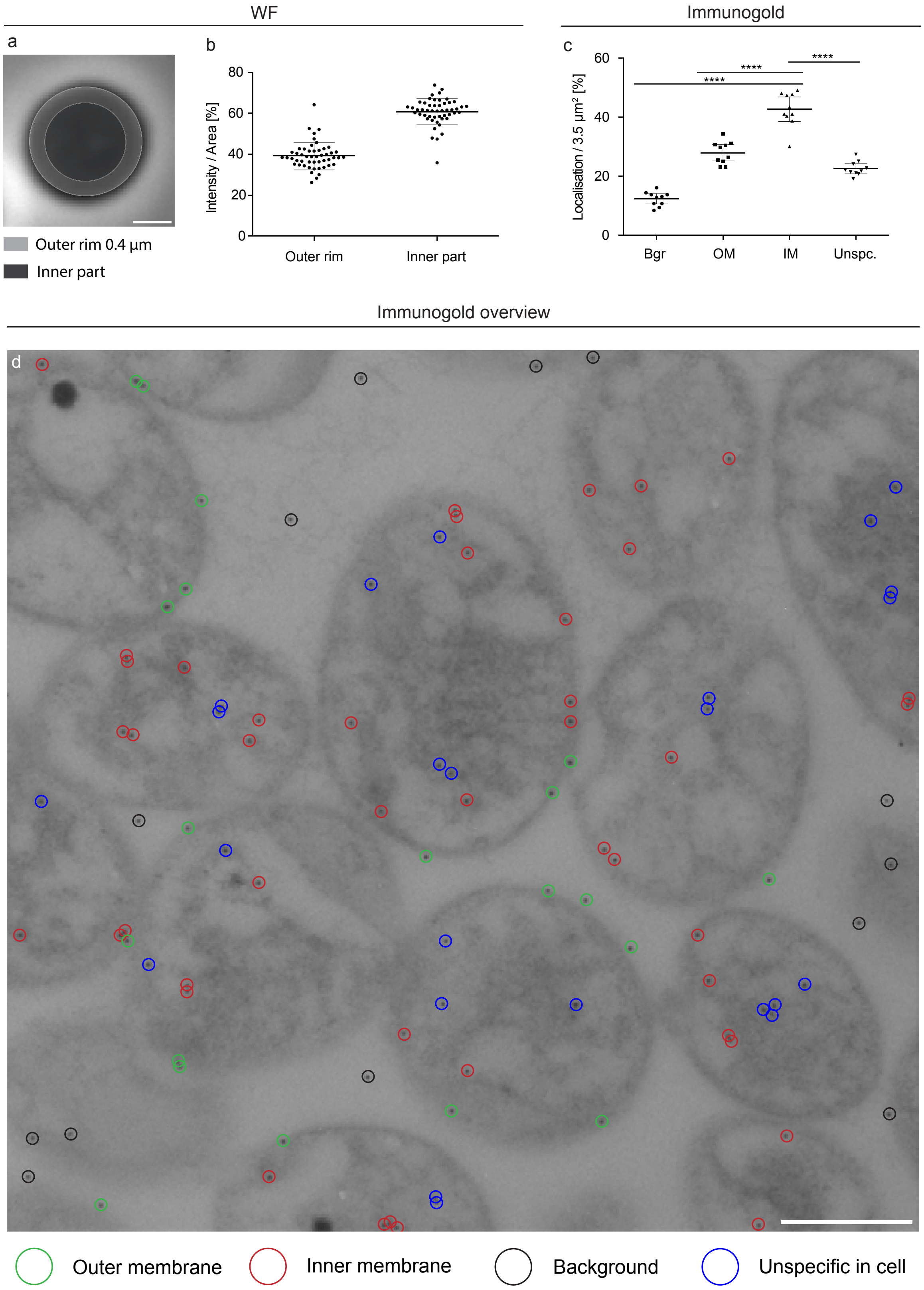
WF
Immunogold
a
Outer rim 0.4 μm
Inner part
b
Intensity / Area [%]
80
60
40
20
0
Outer rim
Inner part
c
Localisation / 3.5 μm² [%]
60
40
20
0
****
****
****
Bgr
OM
IM
Unspc.
Immunogold overview
d
Outer membrane
Inner membrane
Background
Unspecific in cell

Supplementary Figure 5: Quantitative ATPase localisation analysis in *G. obscuriglobus*

(**a+b**) The wide field immunofluorescence quantification was achieved by dividing cells into two parts (**a**). The outer rim comprised a diameter of 0.4 µm and thus covers the entire range where signals associated with the outer membrane (OM) could be expected. In contrast, only invaginations of the cytoplasmic membrane (CM) can cause ATPase signals from the inner part of the cell. (**b**) Comparison of the intensity per area between the inner and outer part of 50 cells in 5 fields of view. 39.21 % of the signals relate to the outer rim and 60.79% to the cell centre. T-test: P=0.0001, error bars indicate 95% confidence interval. Scale bar: 0.5 µm.

(**c**) Analysis of immunogold ATPase labelling of 10 micrographs from three independent experiments. Mean-values are shown and error bars correspond to the 95% confidence interval. T-tests revealed significant differences between localization at the inner membrane compared to background (p=0.0001), to the outer membrane (p=0.0001) and to unspecific signals inside the cells (p=0.0001).

(**d**) Representative micrograph of immunogold labelled ATPase localization in *G. obscuriglobus* cells. Gold particles (black dots) are assigned to four different localizations as shown in Fig. 4c. Diameter of coloured circles indicates uncertainty of localization due to the size of antibodies and gold particle (22.5 nm Ø). Scale bar: 0.5 µm.

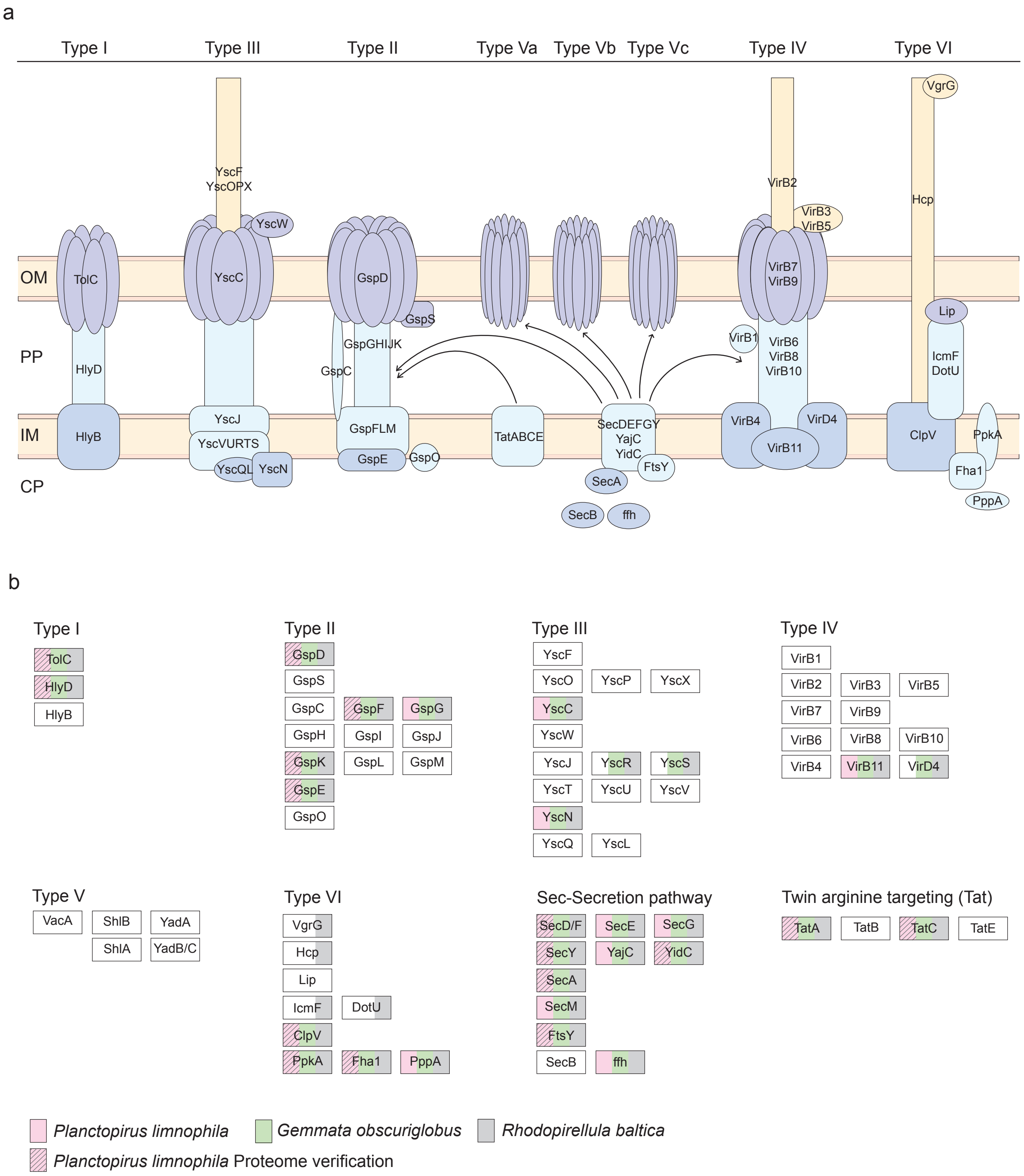
a
Type I
Type III
Type II
Type Va
Type Vb
Type Vc
Type IV
Type VI
OM
PP
IM
CP
TolC
HlyD
HlyB
YscF
YscOPX
YscW
YscC
YscJ
YscVURTS
YscQL
YscN
GspD
GspS
GspGHIJK
GspC
GspFLM
GspE
GspO
TatABCE
SecDEFGY
YajC
YidC
FtsY
SecA
SecB
ffh
VirB2
VirB3
VirB5
VirB7
VirB9
VirB1
VirB6
VirB8
VirB10
VirB4
VirD4
VirB11
VgrG
Hcp
Lip
IcmF
DotU
ClpV
PpkA
Fha1
PppA
b
Type I
TolC
HlyD
HlyB
Type II
GspD
GspS
GspC
GspF
GspG
GspH
GspI
GspJ
GspK
GspL
GspM
GspE
GspO
Type III
YscF
YscO
YscP
YscX
YscC
YscW
YscJ
YscR
YscS
YscT
YscU
YscV
YscN
YscQ
YscL
Type IV
VirB1
VirB2
VirB3
VirB5
VirB7
VirB9
VirB6
VirB8
VirB10
VirB4
VirB11
VirD4
Type V
VacA
ShlB
YadA
ShlA
YadB/C
Type VI
VgrG
Hcp
Lip
IcmF
DotU
ClpV
PpkA
Fha1
PppA
Sec-Secretion pathway
SecD/F
SecE
SecG
SecY
YajC
YidC
SecA
SecM
FtsY
SecB
ffh
Twin arginine targeting (Tat)
TatA
TatB
TatC
TatE
Planctopirus limnophila
Gemmata obscuriglobus
Rhodopirellula baltica
Planctopirus limnophila Proteome verification

Supplementary Figure 6: Planctomycetes lack most proteins of canonical secretion systems

(**a**) Schematic drawing of bacterial secretion systems I to VI, according to the KEGG database bacterial secretion system section.

(**b**) Proteins that correspond to the individual secretion system are indicated in boxes. Homologous proteins encoded in the genomes of *P. limnophila* (pink), *G. obscuriglobus* (green) or *R. baltica* (grey) are indicated in representative colours. In case of *P. limnophila* most predicted proteins were confirmed in proteomic experiments (shaded pink and Supplementary Table 2). While all Planctomycetes possess a slightly modified Sec secretion pathway, only few homologous proteins of the other secretion systems are present. Noteworthy, *R. baltica* harbours almost all genes for a type VI secretion system.

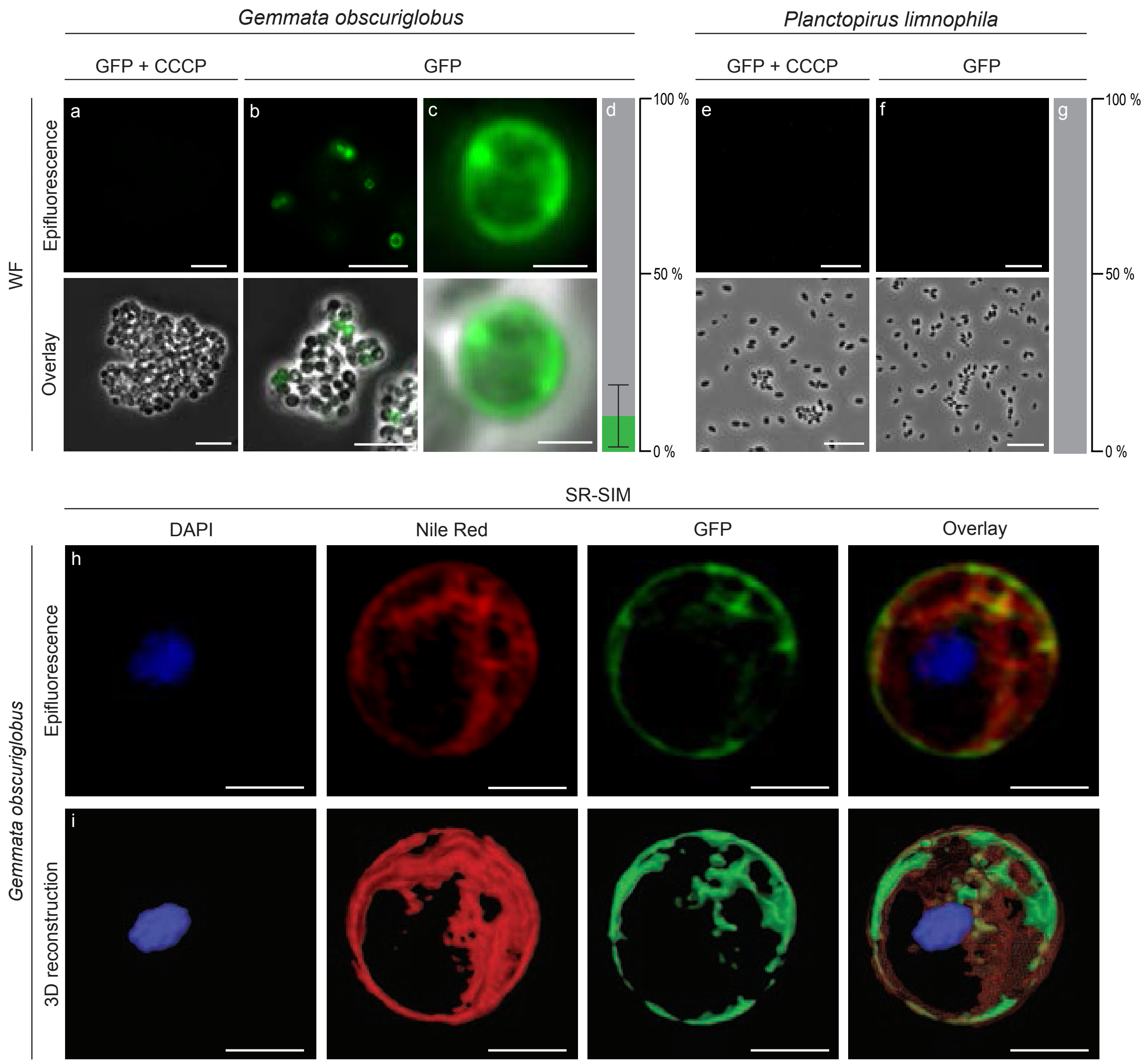
Gemmata obscuriglobus
Planctopirus limnophila
GFP + CCCP
GFP
GFP + CCCP
GFP
WF
Epifluorescence
Overlay
a
b
c
d
e
f
g
100 %
50 %
0 %
100 %
50 %
0 %
SR-SIM
DAPI
Nile Red
GFP
Overlay
h
i
Gemmata obscuriglobus
Epifluorescence
3D reconstruction

Supplementary Figure 7: *G. obscuriglobus* takes up GFP into the periplasm

(**a-d**) *G. obscuriglobus* cells were fed with GFP (green) and analysed with wide field microscopy (WF). Cells poisoned with CCCP showed no signal (**a**), while some untreated cells took up GFP (**b**). Magnified cells revealed regions of intense fluorescence, indicating locally concentrated GFP (**c**). For quantification, among 6 fields of view 1281 cells were counted. 10.2% of the cells internalized GFP (**d**). Error bars indicate standard deviation. Scale bars: 1 µm.

(**e-f**) *P. limnophila* cells do not show GFP uptake in similar experiments. For quantification among 6 fields of view 1294 cells were counted while not a single one internalized GFP (**f+g**). Scale bars: 10 µm.

(**h+i**) *G. obscuriglobus* cells were fed with GFP while membranes were stained with Nile Red and the nucleoid with DAPI (blue). Super-resolution structured illumination microscopy (SR-SIM) revealed an association of the GFP signal with the lipid staining (**h**). The red signal illustrates complex invaginations of the cytoplasmic membrane while the GFP signal corresponds to part of the enlarged periplasmic space in the overlay and the three-dimensional reconstruction (**i** and Supplementary Movie 2). Scale bars: 1 µm.

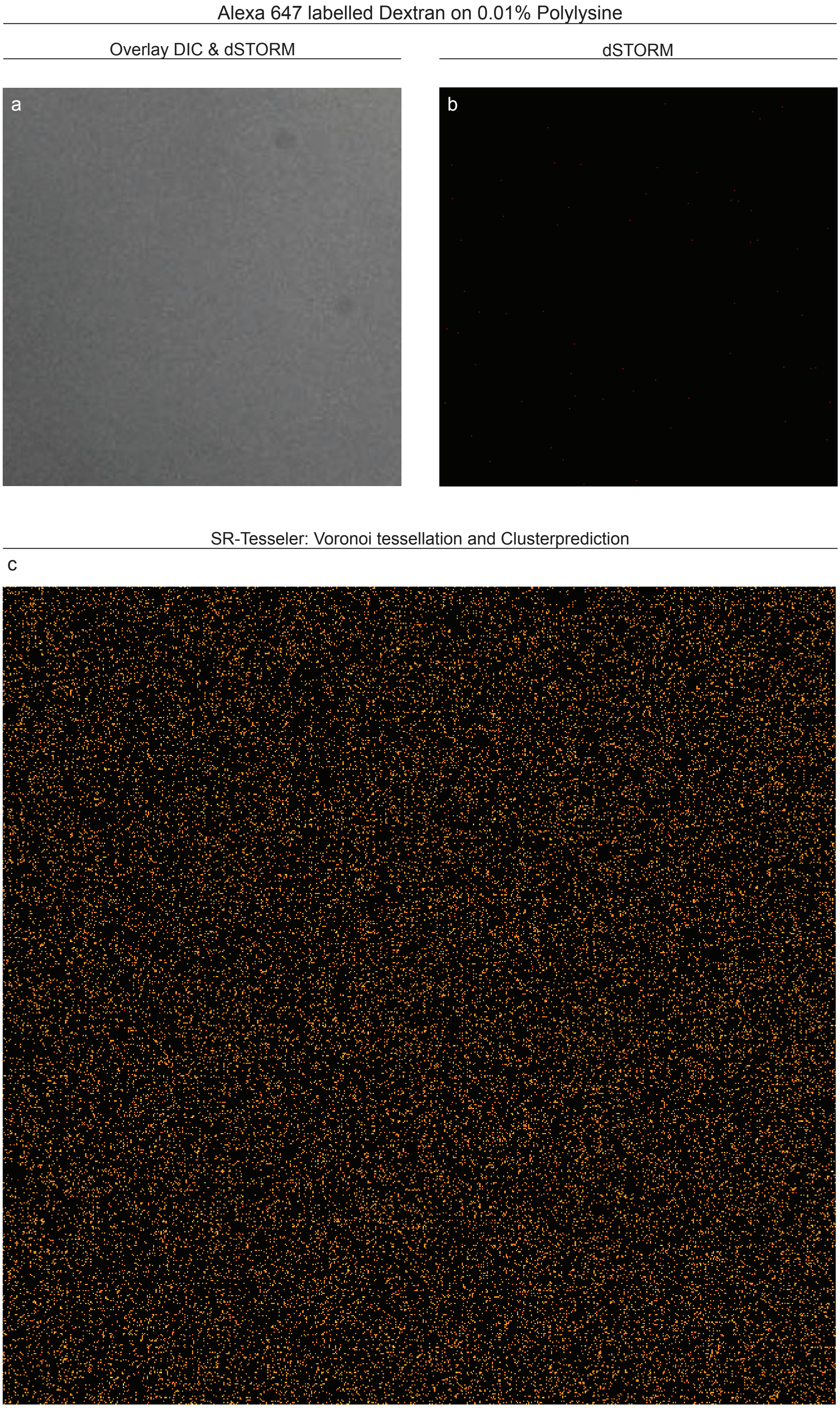
Alexa 647 labelled Dextran on 0.01% Polylysine
Overlay DIC & dSTORM
dSTORM
a
b
SR-Tesseler: Voronoi tessellation and Clusterprediction
c

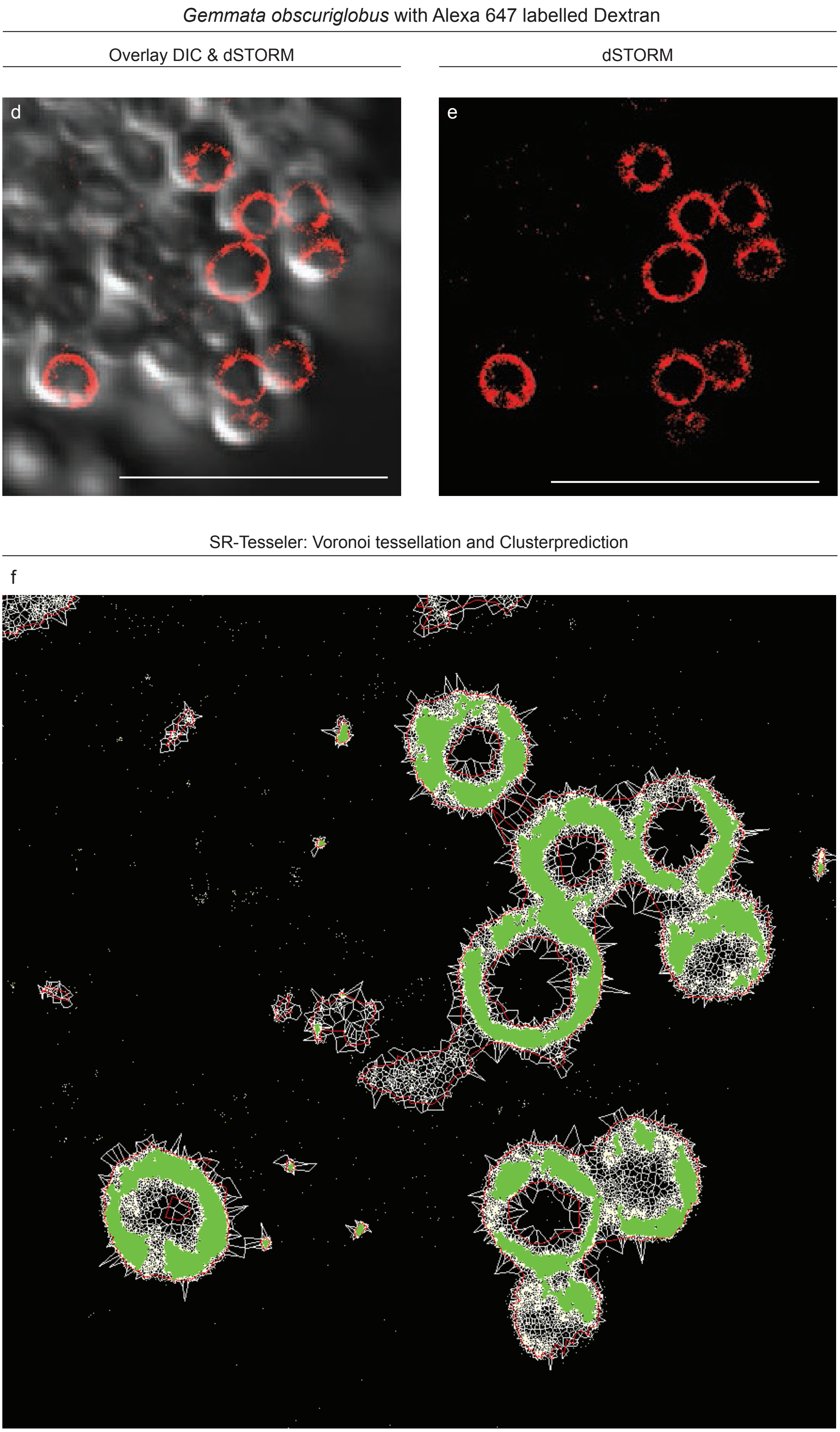

Gemmata obscuriglobus with Alexa 647 labelled Dextran
Overlay DIC & dSTORM
dSTORM
d
e
SR-Tesseler: Voronoi tessellation and Clusterprediction
f

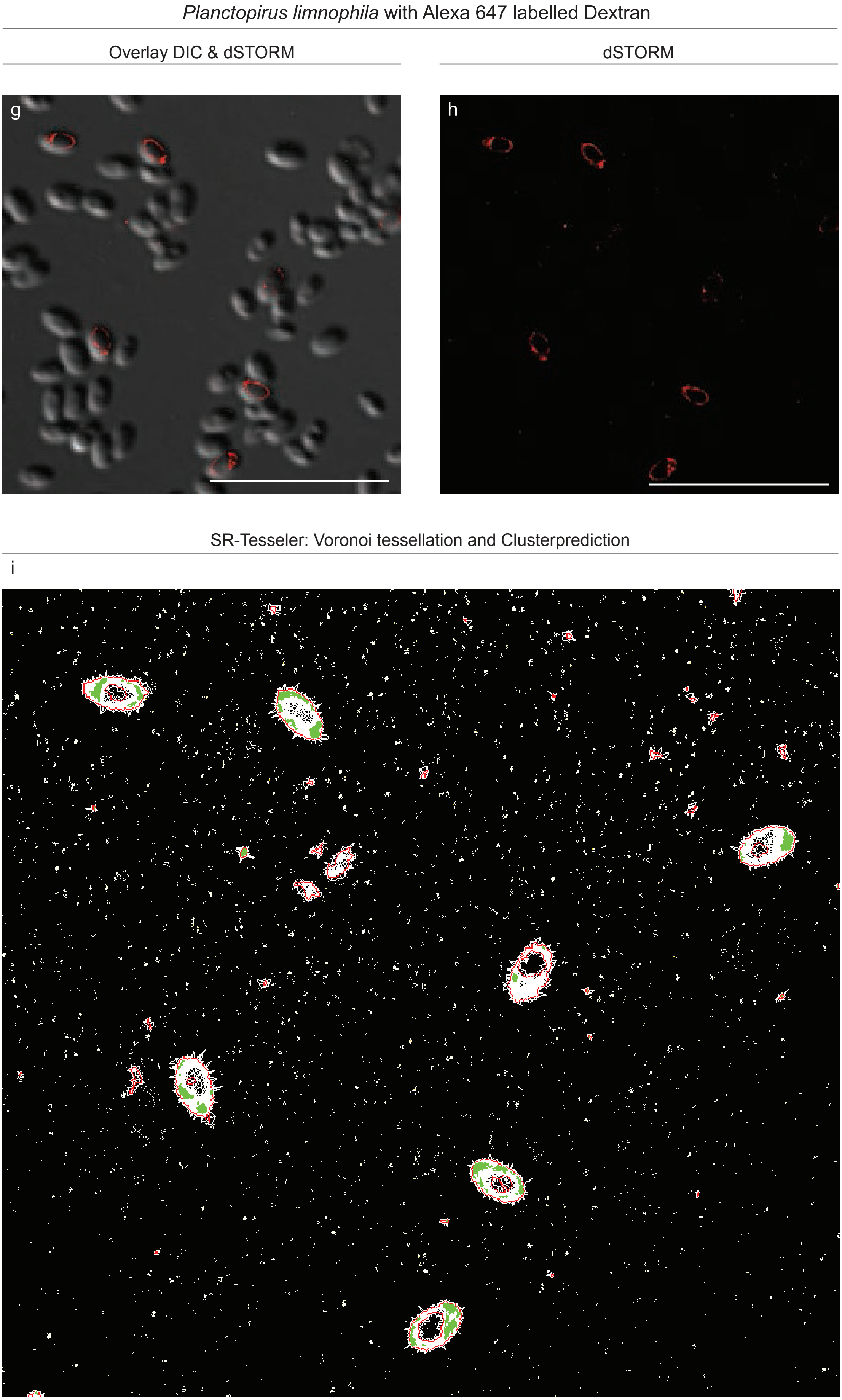
Planctopirus limnophila with Alexa 647 labelled Dextran
Overlay DIC & dSTORM
dSTORM
g
h
SR-Tesseler: Voronoi tessellation and Clusterprediction
i

Supplementary Figure 8: Detailed dSTORM dextran uptake analysis.

(**a-c**) As negative control Alexa Fluor 647 labelled dextran was imaged with differential interference contrast (**a**, DIC) and Super-resolution direct stochastic optical reconstruction microscopic (**b**, dSTORM). No clusters >40 nm were detected with SR-Tesseler applying a minimum of 20 detections.

(**d-f**) Detailed analysis of the dSTORM dextran feeding experiments with *G. obscuriglobus* cells (see Fig. 5e for details). The overlay of a dSTORM and a DIC image demonstrates that red dextran signals localize within the cells (**d+e**). Clusters >40 nm were calculated with SR-Tesseler applying a minimum of minimum 20 detections and results are visualized in green (**f**). Scale bars: 10 µm.

(**g-i**) Detailed analysis of the dSTORM dextran feeding experiments with *P. limnophila* cells (see Supplementary Fig. 9e for overview). The overlay of dSTORM and DIC images demonstrates that the red dextran signals localize within the cells (**g+h**). Clusters >40 nm were calculated with SR-Tesseler applying a minimum of minimum 20 detections and results are visualized in green (**i**). Scale bars: 10 µm.

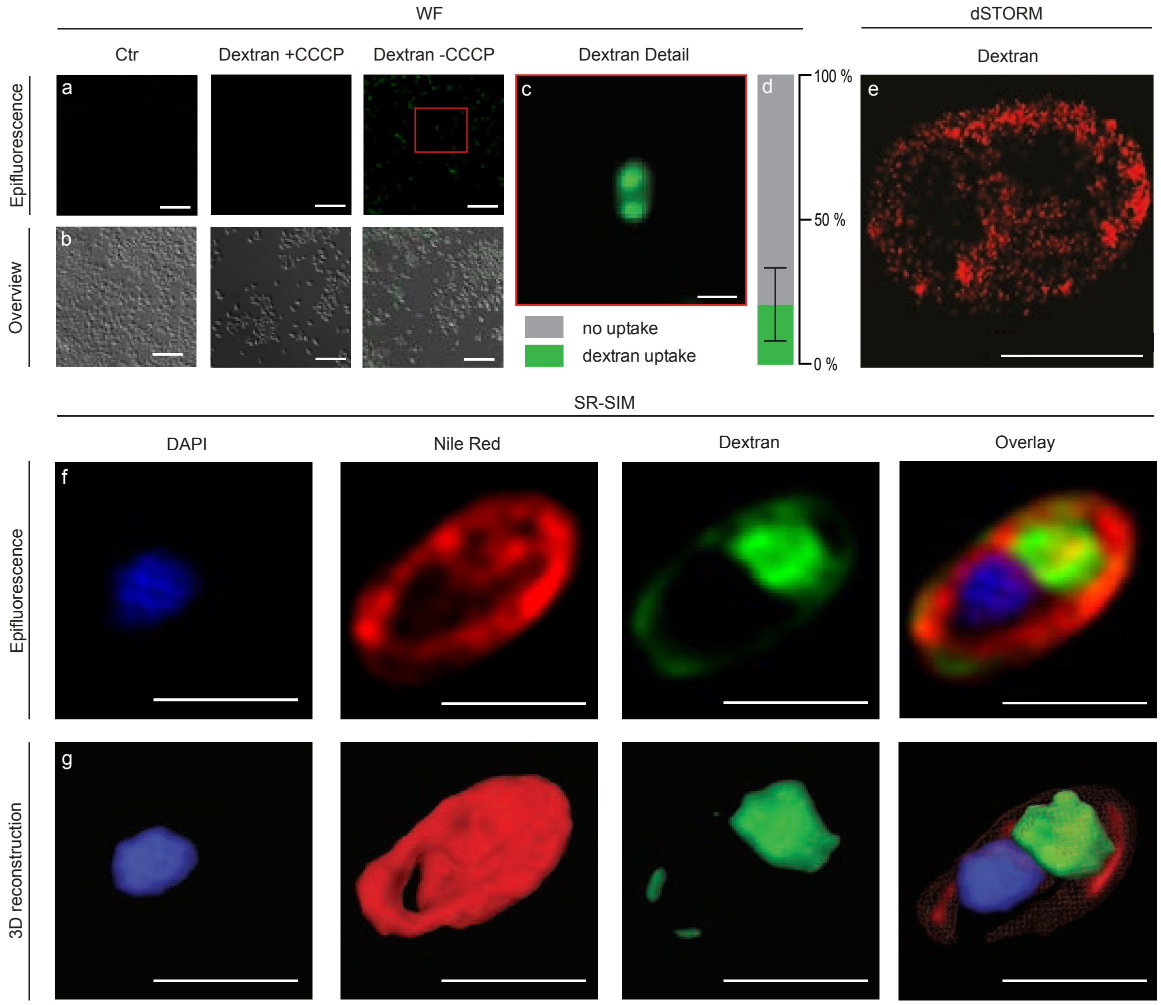

WF
dSTORM
Ctr
Dextran +CCCP
Dextran -CCCP
Dextran Detail
Dextran
a
b
c
d
e
Epifluorescence
Overview
100 %
50 %
0 %
no uptake
dextran uptake
SR-SIM
DAPI
Nile Red
Dextran
Overlay
f
g
Epifluorescence
3D reconstruction

Supplementary Figure 9: *P. limnophila* takes up dextran into the periplasm

(**a-d**) *P. limnophila* cells, except those of the negative control (Ctr), were fed with fluorescein-labelled 40 kDa dextran (green). (**a+b**) Poisoned cells showed no signal (+CCCP), while other cells (-CCCP) took up dextran. Scale bars: 10 µm. (**c**) Magnified cells revealed foci of intense fluorescence, indicating locally concentrated dextran. Scale bar: 1 µm. (**d**) For quantification, among 24 fields of view 15,557 cells were counted and 22.17% of the cells internalized dextran. Error bars indicate standard deviation.

(**e**) Super-resolution direct stochastic optical reconstruction microscopic (dSTORM) analysis of *P. limnophila* cells fed with Alexa 647-labelled 10 kDa dextran. In contrast to diffraction limited micrographs (**c**), no distinct foci are visible, but the red fluorescence signal shows an almost homogeneous distribution of dextran in a confined compartment of the cell (see Supplementary Fig. 8g-I for details). Scale bar: 1 µm.

(**f+g**) *P. limnophila* cells were fed with fluorescein-labelled 40 kDa dextran (green), while membranes were stained with Nile Red and the nucleoide with DAPI (blue). Super-resolution structured illumination microscopy (SR-SIM) revealed that the green dextran signal is localized in invaginations of the inner membrane (red) that enlarge the periplasmic space as shown in the overlay image and in the three-dimensional reconstruction (**g** and Supplementary Movie 4). Scale bar: 1 µm.

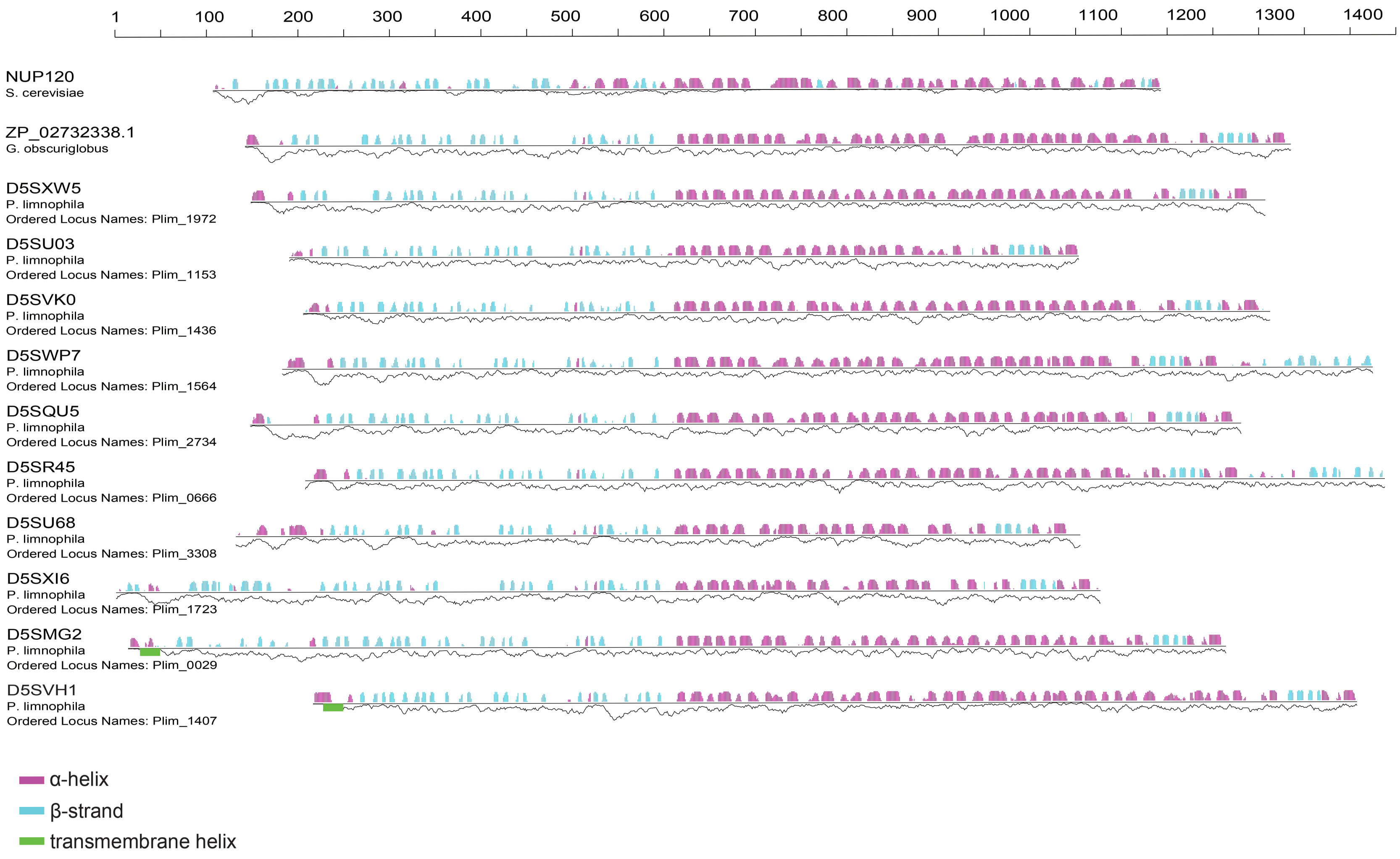

1
100
200
300
400
500
600
700
800
900
1000
1100
1200
1300
1400
NUP120
S. cerevisiae
ZP_02732338.1
G. obscuriglobus
D5SXW5
P. limnophila
Ordered Locus Names: Plim_1972
D5SU03
P. limnophila
Ordered Locus Names: Plim_1153
D5SVK0
P. limnophila
Ordered Locus Names: Plim_1436
D5SWP7
P. limnophila
Ordered Locus Names: Plim_1564
D5SQU5
P. limnophila
Ordered Locus Names: Plim_2734
D5SR45
P. limnophila
Ordered Locus Names: Plim_0666
D5SU68
P. limnophila
Ordered Locus Names: Plim_3308
D5SXI6
P. limnophila
Ordered Locus Names: Plim_1723
D5SMG2
P. limnophila
Ordered Locus Names: Plim_0029
D5SVH1
P. limnophila
Ordered Locus Names: Plim_1407
α-helix
β-strand
transmembrane helix

Supplementary Figure 10: Putative membrane coat-like proteins of *Planctopirus limnophila*

P. limnophila homologs to the MC-like protein ZP_02732338.1 of *G. obscuriglobus* were identified by sequence analyses and secondary structure prediction. The black horizontal lines represent the sequence of each protein. The predicted α-helices (magenta) and β-strands (cyan) are indicated by bars above each line. The height of the bars is proportional to the confidence of the prediction (Psipred). Predicted transmembrane helices are shown in green boxes below the sequences (TMMH). The estimated level of globularity is indicated by a line below the sequence (pred), the higher the line, the more globular. Plim_1972 is the most similar *P. limnophila* homolog to ZP_02732338.1 in terms of sequence comparison and structure prediction.

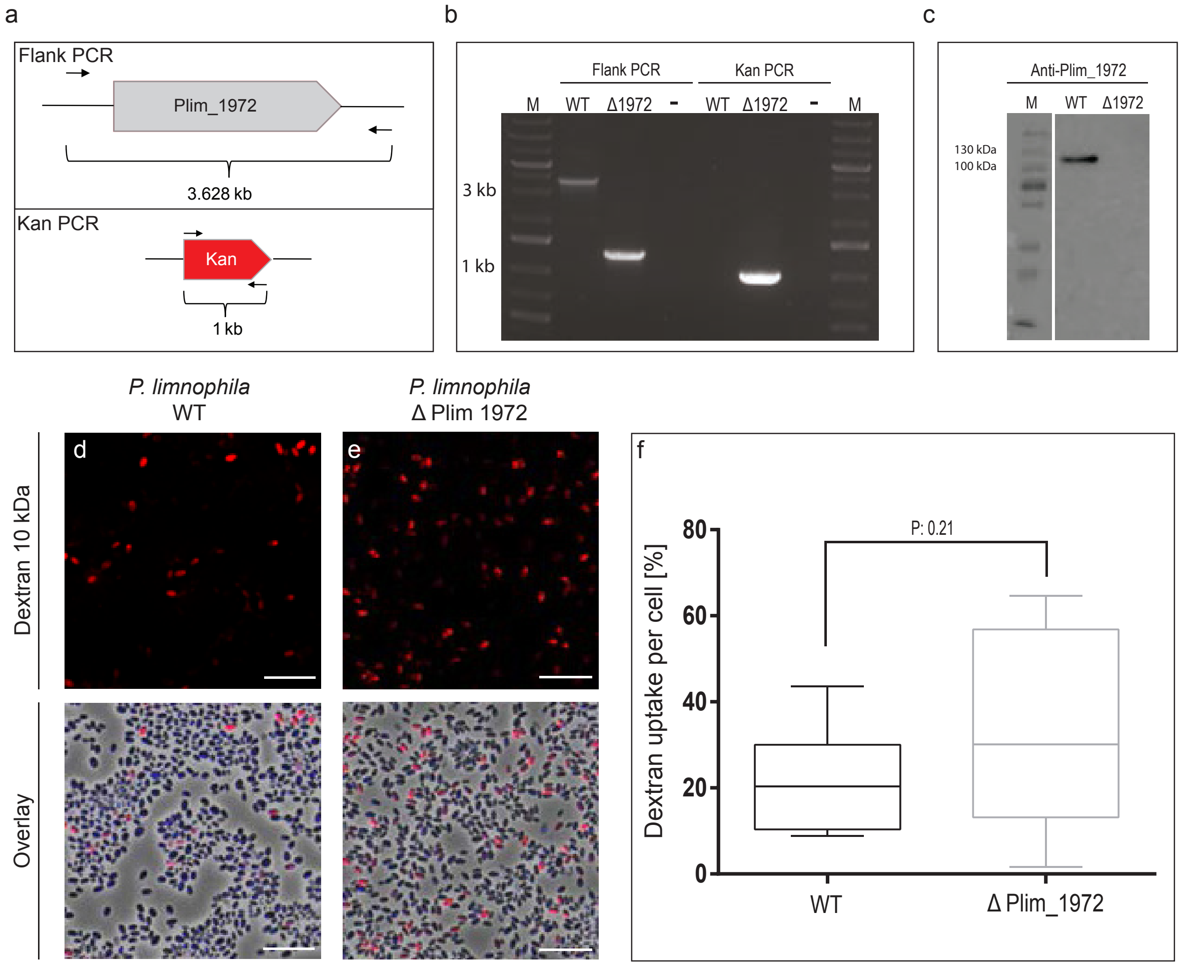
a
Flank PCR
Plim_1972
3.628 kb
Kan PCR
Kan
1 kb
b
Flank PCR
Kan PCR
M WT Δ1972 - WT Δ1972 - M
3 kb
1 kb
c
Anti-Plim_1972
M WT Δ1972
130 kDa
100 kDa
P. limnophila WT
P. limnophila Δ Plim 1972
Dextran 10 kDa
Overlay
d
e
f
P: 0.21
Dextran uptake per cell [%]
80
60
40
20
0
WT
Δ Plim_1972

Supplementary Figure 11: Uptake of dextran in *Planctopirus limnophila* does not require the MC-like protein Plim_1972

(**a**) The Plim_1972 gene of *P. limnophila* was replaced by a kanamycin resistance cassette (Kan). The control PCRs for the mutant validation consisted of two different primer sets. In WT cells the flanking primer set shall amplify a 3.628 kb fragment while the Kan-primer set should not result in any product. In the ΔPlim_1972 mutant, the flanking primer set should result in a 1.1 kb fragment while the Kan-primer set should amplify a 1 kb region of the mutant's genome.

(**b**) PCR products for *P. limnophila* WT cells and the ΔPlim_1972 mutant correspond to the predicted fragment length (**a**), indicating integrity of the mutants' genotype.

(**c**) Western blot analysis of *P. limnophila* WT and the ΔPlim_1972 mutant. The antibody against Plim_1972 detects a protein band around 120 kDa in WT cells while the ΔPlim_1972 mutant shows no respective band. Employing the protein molecular weight calculator (www.sciencegateway.org) a mass of 120.82 kDa was estimated for Plim_1972 which fits to the size of the detected band for WT cells.

(**d-f**) *P. limnophila* WT (**d**) and ΔPlim_1972 mutants (**e**) were fed with fluorescence-labelled 10 kDa dextran. Wide field epifluorescence and epifluorescence / phase contrast overlays demonstrate that both WT and mutant cells take up dextran (red). No significant differences in the uptake among three independent experiments could be detected while 15,556 WT and 19,502 mutant cells were analysed (**f**, T-test: P=0.21, error bars indicate standard deviation). Thus, the protein Plim_1972 is not required for dextran uptake.

Supplementary Table 1: Immunogold localisation of ATPases

Gold particles were counted in 10 fields of view (fov) by three different researchers (R#1-R#3). Localisation was differentiated between background, outer membrane, inner membrane and unspecific within a cell. Thereby a localization uncertainty of 22.5 nm Ø caused by antibody labelling was acknowledged.

fov	Background			Outer Membrane			Inner Membrane			Unspecific in cell		
	R#1	R#2	R#3	R#1	R#2	R#3	R#1	R#2	R#3	R#1	R#2	R#3
1	5	15	12	32	20	17	29	44	57	35	24	13
2	10	10	18	37	30	24	21	49	53	32	21	22
3	4	18	17	36	32	25	33	55	59	23	22	19
4	6	24	18	34	25	30	23	54	67	26	19	18
5	5	15	21	44	44	15	23	52	67	28	17	20
6	6	12	10	42	20	13	26	45	52	23	21	24
7	7	9	16	34	20	22	31	41	49	37	11	14
8	5	7	13	30	21	18	26	40	50	36	23	23
9	6	16	21	34	34	24	22	58	63	33	8	16
10	4	15	22	26	26	27	13	30	47	41	9	16

Supplementary Table 2: *P. limnophila* proteome analysis

List of typical Gram-negative proteins identified by our proteomic approach compared to our secretion pathway prediction (SECPATH, Supplementary Fig. 6) and to the computational analysis by Speth *et al.* 2012[1]. Localization prediction was achieved with PSORTB 3.0.

ACCESSION NUMBER	ANNOTATION	LOCALISATION **WITH** PSORTB 3.0	SECPATH	SPETH *ET AL.*, 2012[1]
ADG68672.1	Putative TolC	Outer membrane	+	+
ADG69500.1	GspD	Outer membrane	+	+
ADG66639.1	Type II/III ss homologue	Outer membrane		+
ADG67415.1	Beta barrel transporter	Outer membrane		+
ADG68191.1	BamA	Outer membrane		+
ADG68627.1	Beta barrel transporter	Outer membrane		+
ADG69297.1	TolC homologue	Outer membrane		+
ADG69651.1	CpaC	Outer membrane		+
ADG65956.1	LptD	Outer membrane		
ADG66250.1	Putative fimbrial subunit	Outer membrane		
ADG66422.1	Hypothetical protein	Outer membrane		
ADG66532.1	Hypothetical protein	Outer membrane		
ADG67744.1	OMPT	Outer membrane		
ADG68522.1	Carboxylase	Outer membrane		
ADG68596.1	Cytochrome C	Outer membrane		
ADG67045.1	Hypothetical protein	Outer membrane		
ADG67290.1	Hypothetical protein	Outer membrane		
ADG67378.1	Glycanotransferase	Outer membrane		
ADG69934.1	Hypothetical protein	Outer membrane		
ADG67060.1	Sulfatase	Outer membrane		
ADG67760.1	BamB	Outer membrane		
ADG69252.1	OMPA	Outer membrane		
ADG69913.1	Porin	Outer membrane		
ADG69212.1	Esterase	Outer membrane		
ADG66736.1	Porin	Cytoplasm		+
ADG69997.1	GspE/PilB	Cytoplasm	+	

ADG67874.1	ClpV/VasG/ClpB	Cytoplasm	+	
ADG68194.1	SecA	Cytoplasm	+	
ADG69980.1	FtsY	Cytoplasm	+	
ADG66008.1	GspF/PilC/PulF	Cytoplasmic membrane	+	
ADG69480.1	PpkA	Cytoplasmic membrane	+	
ADG67183.1	SecD/F	Cytoplasmic membrane	+	
ADG66345.1	SecY	Cytoplasmic membrane	+	
ADG67540.1	YidC	Cytoplasmic membrane	+	
ADG68547.1	TatA	Cytoplasmic membrane	+	
ADG68503.1	TatC	Cytoplasmic membrane	+	
ADG66306.1	Cell adhesion	Extracellular		+
ADG67429.1	HlyD	Periplasm	+	
ADG69451.1	GspK/ PulK	Periplasm	+	
ADG66488.1	Putative Secretin	Unknown		
ADG70027.1	Fha1	Unknown	+	
ADG67044.1	TolC homologue	Unknown		
ADG67856.1	OMP	Unknown		+

Supplementary References

1. Speth DR, van Teeseling MC, Jetten MS. Genomic analysis indicates the presence of an asymmetric bilayer outer membrane in planctomycetes and verrucomicrobia. *Frontiers in microbiology* **3**, 304 (2012).
